# A Systematic Review of the Diagnostic Methods of Small Fiber Neuropathies in Rehabilitation

**DOI:** 10.3390/diagnostics10090613

**Published:** 2020-08-20

**Authors:** Rita Chiaramonte, Marcello Romano, Michele Vecchio

**Affiliations:** 1 Department of Biomedical and Biotechnological Sciences, Section of Pharmacology, University of Catania, 95125 Catania, Italy; 2 Neurology Unit, Azienda Ospedaliera Ospedali Riuniti Villa Sofia Cervello, 90100 Palermo, Italy; mc.romano1958@gmail.com; 3 Rehabilitation Unit, AOU Policlinico Vittorio Emanuele, University of Catania, 95125 Catania, Italy

**Keywords:** small fiber neuropathy, diagnosis, systematic reviews

## Abstract

This systematic review describes the several methods to diagnose and measure the severity of small fiber neuropathies and aims to guide the physician to define all the diagnostic approaches for adopting the best strategies described in the current literature. The search was conducted in PubMed, EMBASE, Cochrane Library and Web of Science. Two reviewers independently reviewed and came to consensus on which articles met inclusion/exclusion criteria. The authors excluded all the duplicates, animals’ studies, and included the English articles in which the diagnostic measures were finalized to assess the effectiveness of rehabilitation and pharmacologic treatment of patients with small fiber neuropathies. The search identified a total of 975 articles with the keywords “small fiber neuropathy” AND “rehabilitation” OR “therapy” OR “treatment”. Seventy-eight selected full-text were analyzed by the reviewers. Forty-one publications met the inclusion criteria and were included in the systematic review. Despite the range of diagnostic tools for the assessment of small fiber neuropathy, other robust trials are needed. In addition, always different diagnostic approaches are used, a unique protocol could be important for the clinicians. More research is needed to build evidence for the best diagnostic methodologies and to delineate a definitive diagnostic protocol.

## 1. Introduction

### Small Fibre Neuropathy

Small fiber neuropathy (SFN) is caused by impairment of unmyelinated C and thinly myelinated Aδ fibers. The symptoms are characterized by sensory symptoms, pain and autonomic symptoms, such as palpitations, gastrointestinal disturbances, and orthostatic dizziness. The symptoms and signs can be present as spontaneous (e.g., burning, deep, itching and paroxysmal) or evoked (e.g., thermal allodynia, light tough allodynia and hyperalgesia) pain.

The diagnostic criteria for small fiber neuropathy are not established, influencing the approach to patients in clinical practice [1].

Nerve biopsy, genetic testing and quantitative sensory testing (QST) permit the definitive diagnosis, due to limitation of clinical examination, needle electromyography and nerve conduction.

The sensitivity (78–92%) and specificity (65–90%) of skin biopsy for diagnosing a SFN is high [2].

The punch skin biopsies could identify decreased intra-epidermal nerve-fiber density (IENFD) of unmyelinated nerves. QST could be a useful screening test for small and large fiber neuropathies [3]. The combination of clinical signs and abnormal QST and/or IENFD findings is a more reliably diagnostic method for SFN than the combination of abnormal QST and IENFD findings in the absence of clinical signs [1]. Sensory symptoms alone should not be considered a reliable screening feature [1]. Physical examination often does not reveal any abnormalities because muscle strength, vibration sense and tendon reflexes are often preserved. Moreover, QST is unable to distinguish between central and peripherical disorders and feigned and true loss of sensation and, moreover it requires the collaboration and conscious integration from the patient [4].

Laser-evoked potentials (LEPs) are another reliable diagnostic method to assess the Aδ-fiber, sensible to physiological differences, such as body height, age, gender and stimulation site [5]. Somatosensory evoked potentials are neurophysiological methods that assess function of or small fibers and are neurophysiological methods that assess function of large or small fibers. Among nociceptive mediated evoked potentials, contact heat evoked potentials (CHEPs) are also commonly used for investigating patients with neuropathic pain [6]. Microneurography records the nociceptive activity of C-fibers [7]. The ultrasonography in small fiber neuropathies, in addition, reveal an enlarged cross-sectional area similar to that in large fiber polyneuropathy, which eases the placement of microneurography [8]. Ultrasound, in combination with an ‘open access’ amplifier and data capture system (Open-Ephys), simplifies the procedure of microneurography [9]. The nociceptive flexion reflex (RIII) is a widely investigated neurophysiological tool for the clinical evaluation of neuropathic pain [10]. Learned strategies for RIII suppression without presentation of feedback and the RIII feedback training gave encouraging results [11]. The cutaneous silent period (CuSP), a transient suppression of electromyographic voluntary activity that follows painful stimuli, could serve as an objective functional measure of the δ fibers in peripheral neuropathies, it is simple and well tolerated [12]. Laser Doppler flowmeter (LDF) and laser Doppler imaging (LDI) permit us to analyze vasomotor small fiber function by quantifying the integrity of the C-mediated local axonal reflex [13,14].

The nerve conduction study, in support of a probable pure SFN, could participate in the diagnostic work-up excluding other neuropathy, in fact in the SFN there are normal sural nerve action potential amplitude and conduction velocity findings [15,16].

Genetic tests are useful for specific diagnosis of SFN, genetic and laboratory tests, such as nerve conduction study are useful for exclusion diagnosis [16].

Sudomotor dysfunction is often an early symptom of autonomic dysfunction in SFN [17]. Sudomotor axon reflex testing (QSART) and sudoscan could be an important tool for a precocious diagnosis [18]. QSART is abnormal in >70% of SFN [17].

Measurement of decreased intra-epidermal nerve fiber density (IENFD) of unmyelinated nerves in punch skin biopsy specimens is a well-validated and highly reproducible diagnostic biomarker of SFN [19,20].

Corneal confocal microscopy detects greater corneal nerve fiber loss in patients with painful neuropathy and this correlates with the severity of neuropathic pain [21].

Several scales are not specific for SFN, and most scores measure better large than small fiber neuropathies. Several scales quantify the symptomatology, especially the neuropathic pain, such as the McGill pain questionnaire, neuropathy impairment score (NIS), neuropathic pain scale (NPS), numeric rating scale (NRS), visual analogue scale (VAS), and pain detection questionnaire (PD-Q9) [22].

The neuropathy impairment score and the Michigan Diabetic Neuropathy Score (MNDS) demonstrated a weak but significant association with QSART in the foot, which is a measure of SFN [23].

Other tests to diagnose SFN in Sjögren’s Syndrome (SS) require more invasive approaches (i.e., sural-nerve biopsies) [19].

A multidisciplinary assessment of SFN is very important to reduce the disability. The importance of identifying the severity of the symptoms and the modifications during rehabilitation and pharmacologic therapy has important implications for management.

Neuropathic symptoms have a negative impact on the quality of life [24].

Our systematic review defined the several methods to assess SFN and to guide the physician to delineate a diagnostic protocol adopting the best strategies described in the current literature. Our guide could help the multidisciplinary team to measure, objectively and easily, the severity of SFN and to assess the disorder. The current literature did not describe a unique diagnostic protocol and use arbitrarily, several methods. A diagnostic protocol should make this more objective, reproducible, and repeatable by the multidisciplinary team.

## 2. Methods

### 2.1. Search Strategy

The search was carried out on the following medical electronic databases: PubMed, EMBASE, Cochrane Library and Scopus Web of Science. The review was conducted from 22 May to 1 July 2020.

### 2.2. Selection Criteria and Data Extraction

**Figure 1:**
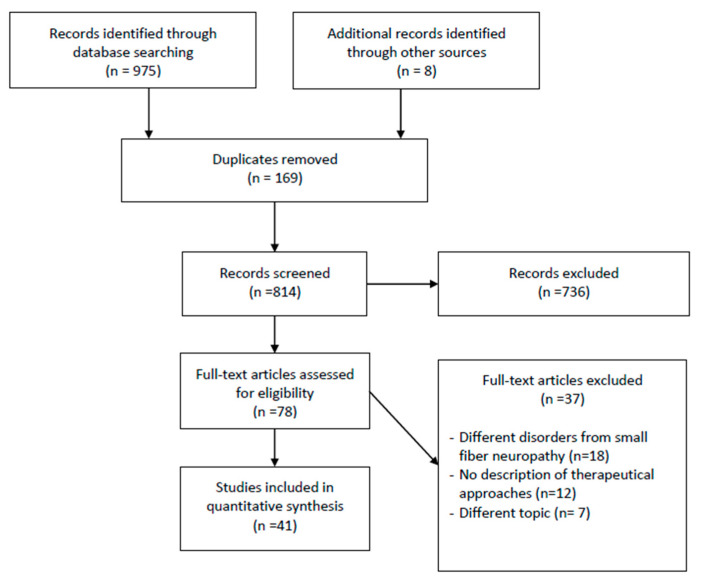
Flowchart of the process of literature search and extraction of studies meeting the inclusion criteria.

Studies considered for this review have to include the diagnostic methods in patients with SFN. We included English original articles about diagnostic tools useful to determine the severity of SFN after therapy. We excluded animal studies, participants with other neuropathies. We also excluded all of the remaining duplicates (Figure 1).

Two reviewers (C.R. and V.M.) independently screened the titles and abstracts from the initial search to identify relevant records and to identify eligible studies based on title and abstract. Selected full texts were then reviewed and included in the systematic review, following the PRISMA protocol [25] and in accordance with the PICOS criteria [26] (population, intervention, comparison, outcome, and study design) shown in Table 1: Participants were all patients affected by SFN; intervention was based on rehabilitation therapy or pharmacological approaches; the comparator was any comparator; the outcomes included clinical assessments, diagnostic scales, electromyography and nerve conduction, and biopsy; the study design was randomized controlled trial (RCTs), case series and case report retrospective studies.

## 3. Results

### 3.1. Description of the Studies

From 1984 to 2019, the database searched of 975 articles with the keywords “small fiber neuropathy” AND “rehabilitation” OR “therapy” OR “treatment”, whose titles and abstracts were screened by the reviewers. The papers remained for full text screening were 78 and the eligibility of the study inclusion was assessed independently. Forty-one publications met the inclusion criteria and were included in the systematic review. Thirty-seven were excluded for the following reasons: 18 involved individuals with different disorders from SFN, 7 examined different topics from our aim, 12 did not present any therapeutic procedure (Figure 1).

The qualitative information synthesis for each parameter was attributed to the following evidence levels according to the recommendations of the Oxford Centre for Evidence-Based Medicine: evidence from systematic review of randomized controlled trials (1a), clinical controlled studies (2a), case-control-studies (3a) and from non-systematic reviews [4] (Table 1).

### 3.2. Variations of Experimental Conditions across the Studies

The selected 41 articles were described on the basis of the several diagnostic methods used in each study for the assessment of SFN. Characteristics of the studies are shown in Table 1.

All study groups were not homogeneous for relevant general clinical features as clinical presentation, duration of disease and of the symptoms, kinds of diagnostic measures, severity of symptoms, time of starting therapy, duration of treatment, the follow-up period at the end of the therapy (Table 1).

### 3.3. Diagnostic Examination

We showed all the methods used for the diagnosis of SFN, found in the current literature.

Most of the selected articles for the review were used skin biopsy for the definitive diagnosis and/or genetic tests [54,57] (Table 1). The skin biopsy was used alone [32,48,62] or in the most cases together with other diagnostic procedure, as nerve conduction examinations [33,41,56] or scales to assess the severity of the neuropathic symptoms (Table 1). Quantitative sensory testing (QST) with vibratory (VDT), cold (CDT), and heat-pain (HP) detection threshold testing were added for the specific diagnosis [30,40,61].

The scales most used to assess neuropathic pain, disability and handicap related to the symptoms were the visual analogue scale (VAS) [43,45,49,50,58,60,61,64,65,66], the numerical rating scale (NRS) [29,31,34,35,36,38,39,40], the neuropathic pain scale (NPS) [34,35,36,43,64], patient’s global impression of change (PGIC) [34,35,36,39,46,63,64], the small fiber neuropathy symptom inventory questionnaire (SFNSIQ) [34,35,36], the generic short form health survey (SF-36) [34,35,36,38], the Rasch-built overall disability outcome scale [34,35,36], verbal rating scale (VRS) [63], the Michigan neuropathy screening instrument (MNSI) symptoms questionnaire [45,58], the neuropathy impairment score (NIS) [28,44,65], the daily sleep interference score (DSIs) [39], and the McGill scores [29,63]. For sarcoidosis SFN, the small-fiber neuropathy screening list (SFNSL) is used [61].

Other tests used in SFN were the sudoscan [52] and the quantitative sudomotor axon reflex testing (QSART) [45,46,53,58,59]. Handicap was evaluated using the modified Rankin scale [55]. 

A complete blood count, electrolytes, calcium, magnesium, creatine kinase, thyroid-stimulating hormone, vitamin B12, haemoglobin A1c, fasting glucose, creatinine, urea, and serum protein electrophoresis [27] could have played a role in excluding other disorders.

### 3.4. Diagnostic Guide and Clinical Consequences

SFN can be idiopathic or associated with other disorders. The symptoms worsen over time, but the progression is typically slow. The diagnostic process is often complex, also due to the differential diagnosis that pathology requires. According to our experiences, and supported by the literature (Table 2), specific scales are essential for quantifying the impairment and assessing the response to therapy and symptom modifications during follow up. QSART and sudoscan are very useful tools, especially at the beginning of the evaluation to evaluate the autonomic symptoms, which are very often present. In the general evaluation, it always seems extremely useful to include threshold and peripheral nerve conduction studies, to better define the characteristic of the SFN and exclude other concomitant causes. Genetic testing and corneal confocal microscopy are often used for diagnostic confirmation. Skin biopsy, simpler than nerve biopsy, is necessary for a definitive diagnosis.

Treatment of SFN certainly depends on the underlying cause, when detectable, but it is often limited to symptomatic therapy, which is also essential for improving adherence to rehabilitation treatment. The duration of treatment is based on the severity of the symptoms and the progression of the disease. It seems important to understand the complexity of this pathology in order to follow an adequate diagnostic procedure and to find the best therapeutic management to limit the progressive worsening of symptoms, which although generally slow is often present, and consequently the reduction in the quality of life.

## 4. Discussion

Our systematic review focused on the several measures useful for the examination of SFN severity after pharmacological or rehabilitative therapy. We realized a comprehensive overview to give a guide to ease the collaboration of a multidisciplinary team.

### Comparing Studies: Diagnostic Tools

The definitive diagnosis is based on biopsy. Nerve conduction reveals no abnormality, but is mandatory as exclusion criteria (Table 1).

To assess the progression or the answer to treatment a lot of scales quantified the neurological symptoms especially the pain and indicated the frequency and severity of neuropathic symptoms: VAS [43,45,49,50,58,60,61,64,65,66], NRS [29,31,34,35,36,38,39,40], PGIC [34,35,36,39,46,63,64], NPS [34,35,36,43,64], SFNSIQ [34,35,36], VRS [63], MNSI symptoms questionnaire [45,58], NIS [28,44,65], DSIs [39], the McGill scores [29,63], and SFNSL [61]. The small-fiber neuropathy screening list (SFNSL) was used by van Velzen et al. [61]. This test is specifically developed and validated for SFN in sarcoidosis [42]. The SFNSL consists of 21 questions related to neuropathic pain and to autonomic dysfunction.

Other scales showed the modification of quality of life the disability, the handicap, such as the SF-36 [34,35,36,38], the DSIs [39], the Rasch-built overall disability outcome scale [34,35,36], the modified Rankin scale by Pereira et al. [55].

Quantitative sudomotor axon reflex testing (QSART) [67] is used by five studies [45,46,53,58,59].

Sudoscan, used by Nevoret et al. [52], is a device is a two-min, painless, non-invasive, quantitative test measuring C-fiber postganglionic sympathetic nerve function to the sweat glands of the palms and soles.

A blood investigation and the electrophysiological studies have the role to exclude other disorders. Anderson et al. [27] examined in their case report the complete blood count, electrolytes, calcium, magnesium, creatine kinase, thyroid-stimulating hormone, vitamin B12, hemoglobin A1c, fasting glucose, creatinine, urea, serum protein electrophoresis. The blood examination was normal.

Favoni et al. [37] assessed the role of antiganglioside antibodies in SFN.

Van Velzen et al. [61] and Hilz et al. [40] used QST. It consists of a battery of psychophysical tests and the patient respond to a specific sensory stimulus to the skin [61]. The tests include cold and arm detection threshold (WDT), cold and warm pain threshold, paradoxical heat sensation, allodynia, and vibration detection threshold. Loss of function (i.e., an increased response threshold) for cold and WDT are indicative of SFN. More objective QST measures include laser-evoked potentials and contact heat-evoked potentials where a short stimulus result in activation of thermo-nociceptive cutaneous nerve fibers [61]. Hilz et al. [40] found that vibratory (VDT), cold (CDT), and heat-pain (HP) detection threshold testing adequately characterized Aß-, Aδ-, and C-fiber dysfunction in Fabry patients. Fewer patients had abnormal results of VDT, CDT, HP, and HP after and before therapy with ERT. The most had always had normal threshold. Van Velzen et al. [61] showed that ARA 290 increases sensory pain thresholds, cold pain threshold and warm pain threshold. Azmi et al. [30] assessed the severity of SFN with vibration perception threshold (VPT), cold threshold (CT), warm threshold (WT), neurophysiology, deep breathing heart rate variability (DB-HRV), intraepidermal nerve fiber density (IENFD), and corneal nerve fiber density (CNFD), branch density (CNBD), and fiber length (CNFL). Gaillet et al. [38] used a quantitative sensory testing at the four extremities with measurement of the average warm detection threshold (WDT) [68]. Namer et al. [51] used temperature thresholds and the genetic examination of the mutation of SFN. Hoitsma et al. [69] used the temperature threshold testing (TTT) for sensory fibers and cardiovascular autonomic testing for autonomic fibers, that resulted abnormal in their case report. In the study of Schiffmann et al. [57], the thermal thresholds remained unchanged after enzyme replacement therapy.

## 5. Conclusions

The diagnosis and the follow up of SFN is indispensable for the improvement of quality of life of the individuals with neuropathic symptoms. SFN has a negative psychosocial impact in the lives of the patients and of their families.

We performed a systematic review of the several methods present in the current literature for an accurate examination of SFN. We showed all the diagnostic methods described in the current literature to diagnose and follow the subjects with SFN. On the basis of the diagnostic methods, the physicians could obtain a guide and a common protocol for a multidisciplinary team. The accurate and repeatable assessments could improve the outcome of therapy approaches too. Our guide should help the multidisciplinary team to collaborate, to compare their own assessments with those of other members of the team, and to have more complete examinations. Despite the range of diagnostic tools for SFN, robust trials miss, and thus, different diagnostic approaches are to be used. More research is needed to build evidence for the best diagnostic methodologies and to delineate a definitive diagnostic protocol.

## Figures and Tables

**Table 1:** Diagnostic methods of small fiber neuropathy (SFN). Characteristics and outcomes of studies included in the systematic review.

Authors, Year	Study Design	Patients. Age	SFN Disease. Age at Onset Diagnosis	Onset SFN Symptoms	Symptoms	Diagnosis	Conclusions
Anderson 2017 [27]	Case report	1 p, 35 yo	SFN associated with hantavirus infection	One m after hantavirus infection	Severe, intractable burning limb pain. Allodynia to light touch and hyperalgesia to pinprick in a stocking distribution up to the mid-calf bilaterally	Complete blood count, electrolytes, calcium, magnesium, creatine kinase, thyroid-stimulating hormone, vitamin B12, haemoglobin A1c, fasting glucose, creatinine, urea, serum protein electrophoresis	At follow-up 4 months later, his limb pain was only marginally improved after gabapentin and naproxen
Apfel 2000 [28]	CT	A: 418 rhNGF B: 461 placebo 18–74 yo	Diabetic SFN	-	Neuropathic pain	Neuropathy Impairment Score, Neuropathy Symptom and Change questionnaire, PBQ	Significant beneficial effect of rhNGF on diabetic polyneuropathy
Aradillas 2015 [29]	Case series	33 p, 45.7 yo	SFN related to CRPS	9.7 y	Neuropathic pain	Skin biopsy, NRS, McGill scores.	PE is effective patients with severe long-standing CRPS
Azmi 2015 [30]	OS	49 p A: 18 CSII 55.4 ± 2.9 y B: 31 MDI 49.9 ± 3.3 y	Diabetic SFN	A: 34.8 ± 3.1 y B: 35.2 ± 3.6 y	Neuropathic pain	VPT, CT, WT, DB-HRV, IENFD, CNFD, CNBD, CNFL	MDI group showed no significant change with continuous Subcutaneous Insulin Infusion, but the CSII group showed an improvement in corneal nerve morphology, consistent with regeneration.
Birnbaum 2018 [31]	OS	23 p, ~53.6 y 44 ± 13 y	SS	49.5 ± 23 yo	Pain. Eleven patients had stocking-and-glove pain, and 12 patients had non-stocking and-glove pain. Ten SFN patients (~45%) had neuropathic pain preceding sicca symptoms.	Punch skin biopsies, NRS	SS SFN had increased frequency of male sex, decreased frequency of multiple antibodies, were frequently treated with opioid analgesics, and could present with non-stocking-and-glove pain
Cao 2015 [32]	Case report	1 p, 36 yo	SFN related to aquagenic pruritus	~ for 3 y after symptoms	Aquagenic pruritus	Skin biopsy	Atenolol is to be preferred to propanolol, in view of its convenient once-a-day dosing and better side effect profile
Dabby 2006 [33]	OS	4 p, ~49 yo	Idiopathic SFN	-	Neuropathic pain. Symptoms were distal and symmetrical in three patients and generalized in one patient	Skin biopsy, normal VPT, CT, WT	Clinical improvement occurred 1–2 w after oral prednisone therapy was initiated.
De Greef 2016 [34]	CT	25 p, 18–80 yo	SCN9A-associated SFN	-	Pain, altered temperature sensation.	NRS, NPS, SFNSIQ, PGIC, SF-36	Lacosamide: a potential treatment option in patients with painful neuropathies, considering the central role of Nav1.7 in pain.
De Greef 2016 [35]	CT	60 p, >18 yo	Idiopathic SFN	-	Pain, altered temperature sensation.	NRS, NPS, DSIS, SFN-RODS, 5-point Likert-scale	Positive findings in SFN after iv IG
De Greef 2018 [36]	CT	24 p 48,3 yo	SCN-SFN	-	Pain and autonomic dysfunction	Skin biopsy, NRS, SF-36, NPS	Significant effect on pain, general wellbeing, and sleep quality after Lacosamide drug
Favoni 2018 [37]	Case report	1 p, 45 yo	Anti-GQ1b antibodies associated with SFN	~2 y after symptoms	Tingling and burning pain sensation in the arms and legs, with nocturnal exacerbation	Antiganglioside antibodies research	Benefit from immunotherapy with Adalizumab
Gaillet 2019 [38]	RS	11 p, 41–62 yo	SS	~6.5 y after symptoms	Pain	NRS, SF-36, warm detection threshold	Efficacy of IVIG treatment for pain relief in SS-SFN with an improvement of quality of life and sensory testing
González-Duarte 2015 [39]	CT	45 p, ~54 y	Prediabetic SFN	-	Neuropathic pain	Skin biopsy, DSIs, PGICs, NRS	Improvement of prediabetic neuropathic pain with pregabalin
Hilz 2004 [40]	OS	22 p, A: 11 p B: 11 p 27.9 ±8 yo C: 25 HC 29 ± 10.4 y	Fabry related SFN	-	Pain	VDT, CDT, HP, TTS, NRS	ERT therapy with agalsidase beta significantly improves function of C-, AΔ-, and Aß- nerve fibers and intradermal vibration receptors in Fabry neuropathy
Hoeijmakers 2016 [41]	CR	2 p, ~15 yo	1 p idiopathic SFN, 1 p diabetic SFN	~7 y after symptoms	Painful itch and tingling of legs, dysautonomia symptoms	Skin biopsy, nerve conduction study	Moderate pain relief with treatment with gabapentin in a case. Treatment with duloxetine, combined with a rehabilitation program, resulted in a marked improvement in daily functioning.
Hoitsma 2006 [42]	OS	1 p, 39 yo	SSFN	-	fatigue, neuropathic pain, autonomic dysfunction, and arthralgia	TTT, cardiovascular autonomic function test	SFN seems not an irreversible disorder, infliximab had good outcomes
Hong 2013 [43]	1 p, 64 yo		Diabetic SFN	~2 y	Peripheral neuropathic pain in his both feet	NPS, VAS	The whole body vibration is a good complimentary treatment
Keohane 2017 [44]	CT	A: 48 B: 44 18–75 yo	Amyloid SNF	-	Distal-to-proximal sensorimotor neuropathy with autonomic symptoms	TTR V30M mutation. Biopsy. NIS-LL	Tafamidis delays neurologic progression in early stage ATTRV30M-FAP.
Kluding 2012 [45]	OS	17 p 58.4 ± 5.98 yo	Diabetic SFN	12.4 ± 12.2 y	Pain	VAS, MNSI, QSART, skin biopsy	Exercises improve SFN symptoms
Liu 2018 [46]	RS	55 p, 41 ± 17	Autoimmune SFN	6.3 ± 6.3 y	Neuropathic pain	QSART, 11-point numeric scale, seven-point PGIC.	IVIg is safe and effective
MacDonald 2019 [47]	RS	87 p	SFN	3.2 y	Neuropathic pain	Skin biopsy	45.5% of patients had at some time been treated with opioid medications for neuropathic pain.
Maino 2017 [48]	Case report	1 p, 74 yo	SFN	~6 y after symptoms	Burning and shooting pain in feet	Skin biopsy	20 m of Dorsal Root Ganglion Stimulation induced a paresthesia covering the entire pain area
Mishra 2012 [49]	Case report	1 p, 22 yo	SFN	~6 m after symptoms	Neuropathic pain	Skin biopsy, nerve biopsy. VAS	Reduction of pain after flupirtine
Morozumi 2008 [50]	OS	5 p, 61.8 y	SSFN	-	Neuropathic pain	VAS, lip biopsy	Beneficial after IVIG therapy
Namer 2019 [51]	Case report	1 p, 69 yo	SNF	~10 y after symptoms	Burning pain	Temperature thresholds	Lacosaminde reduced pain in SFN
Nevoret 2014 [52]	Case report	1 p, 60 yo	CIDP SNP	~2 y	Neuropathic pain	Sudoscan sudomotor function test	Less burning, shooting pains and tingling with IVIG
Parambil 2010 [53]	Case series	3 p	SSFN		Intractable neuropathic pain, autonomic dysfunction	Biopsy, QSART	IVIG appears to be effective in relieving symptoms
Patel 2019 [54]	Case report	1 p, 31 yo	SCN-SNF	~10 y after symptoms	Erythromelalgia, painful flushing and burning paresthesisas of the proximal extremities	Nerve biopsy and genetic testing	Carbamazepine reduced pain
Pereira 2016 [55]	Case series	13 p, 55, yo	SS	~3 y after symptoms	Neuropathic pain, Paresthesia	Modified Rankin Scale	Treatment with corticosteroids with immunosuppressive drugs, as mycophenolate mofetil, had positive results. In contrast, IVIG had disappointing results
Saito 2015 [56]	Case report	1 p, 59 yo	SSFN	10 d	Progressive pain and hypoesthesia of the right lower back associated with fever and constipation	Nerve conduction studies. Skin biopsy	Neurological symptoms were effectively relieved with high-dose steroid therapy
Schiffmann 2006 [57]	CT	25 p, ~34 yo	Fabry disease related SFN	-	Neuropathic pain	Thermal thresholds.	Epidermal nerve fiber regeneration did not occur after enzyme replacement therapy
Smith 2006 [58]	OS	32 p, 60 ± 8.4	Diabetic SFN	7 ± 31 y	Neuropathic pain	Skin biopsy	Rehabilitative exercises improved symptoms
Tavee 2016 [59]	RS	115 p, ~46 yo 62 p IVIG 12 p infliximab 14 p IVIG + infliximab 27 p not treated	SSFN	41 yo	Pain, paraesthesia, dysauthonimc symptoms	Skin biopsy	Beneficial from IVIG and anti-TNF alpha in symptoms
Uyesugi 2010 [60]	Case report	1 p, 80 yo	Keloid related SFN	5 yrs after surgery	Itching, pain, and allodynia	VAS	A SFN related to keloid was treated successfully with botulinum toxin type A.
van Velzen 2014 [61]	CT	A: 12 p B: 13 48,6 yo	SSFN	7 y between the current study and the diagnosis of sarcoidosis	Pain, allodynia, hyperalgesia	SFNSL, VAS, QST, autonomic function testing, skin biopsies or corneal confocal microscopy	Long-lasting beneficial effects of ARA 290
Wakasugi 2009 [62]	Case report	1 p, 40 yo	SSFN	2 m	Paresthesia and burning pain in the distal upper and lower extremities.	Skin biopsy	IVIG therapy was immediately and extremely effective
Walega 2014 [63]	Case report	1 p, 53 yo	BMS related SFN	6,5 m	Bilateral burning pain in the anterior tongue and mucosa of the lips	VRS, PGIC, SF-MPQ2	Positive effects of bilateral stellate ganglion blockade
Weintraub 2009 [64]	CT	A: 90 p 61.1 ± 10.4 B: 104 p 60.6 ± 12.4	Diabetic SNF		Neuropathic pain	VAS, NPS, PGIC	PEMF at this dosimetry was non effective in reducing neuropathic pain
Windebank 2004 [65]	CT	A: 20 p, 58.3 ± 12.2 B: 20 p 62.2 ± 10.7	SFN	>6 m	Painful, distal, symmetrical neuropathy	VAS, NIS	IGF-I was safe, but did not improve symptoms in this 6-month of treatment
Yuki 2018 [66]	Case report	3 p, ~27.3 yo	SFN variant of Guillain-Barre syndrome	The three patients developed the symptoms 42, 6 and 11 d respectively after symptom onset	Pinprick sensation with hyperesthesia and brush allodynia in a glove-and-stocking distribution	Skin biopsy	One patient showed no response to IVIG but good response to prednisolone. One patient had no significant improvement with prednisolone. One patient had gradual spontaneous recovery

Painful small-fiber neuropathies (SFN), patients (p), Sjögren’s syndrome (SS), years (y), years old (yo), observational study (OS), retrospective study (RS), clinical trials (CT), bis in die (b.i.d.), weeks (w), days (d), pain intensity numerical rating scale (NRS), small fiber neuropathy symptom inventory questionnaire (SFNSIQ), patient’s global impression of change (PGIC), short form health survey (SF-36), intravenous (iv), immunoglobulin (IG), neuropathic pain scale (NPS), daily sleep interference scale (DSIS), the short form 36 health survey (SF-36), healthy controls (hc), sarcoidosis-associated small fiber neuropathy (SSFN), patient benefit questionnaire (PBQ), recombinant human nerve growth factor (rhNGF), vibration perception threshold (VPT), cold threshold (CT), heat-pain perception thresholds (HP), warm threshold (WT), deep breathing heart rate variability (DB-HRV), intraepidermal nerve fiber density (IENFD), corneal nerve fiber density (CNFD), corneal nerve branch density (CNBD), corneal nerve fiber length (CNFL), subcutaneous insulin infusion (CSII), daily insulin injection (MDI), complex regional pain syndrome (CRPS), plasma exchange (PE), enzyme replacement therapy (ERT), daily sleep interference score (DSIs), total symptom score (TSS), temperature threshold testing (TTT), neuropathy impairment score NIS, neuropathy impairment score—lower limbs (NIS-LL), chronic inflammatory demyelinating polyneuropathy (CIDP), pulsed electromagnetic field (PEMF), intravenous (iv), small-fiber neuropathy screening list (SFNSL), quantitative sensory testing (QST), burning mouth syndrome (BMS), verbal rating scale (VRS), insulin-like growth factor-I (IGF-I), sudomotor axon reflex testing (QSART), Michigan diabetic neuropathy screening instrument (MNSI).

**Table 2:** Diagnostic methods for SFN, safety and effectiveness.

Diagnostic Methods	Features of Diagnostic Tool	Type of SFN	Effectiveness of Diagnostic Methods	Authors
Corneal confocal microscopy		SSFN	It detects greater corneal nerve fiber loss in patients with painful neuropathy and this correlates with the severity of neuropathic pain (Kalteniece 2018) [21]	van Velzen 2014 [61]
Genetic tests	TTR V30M mutation	Amyloid SNF SCN-SNF	For specific diagnosis of SFN	Keohane 2017 [44] Patel 2019 [54]
Laboratory test		SFN associated with hantavirus infection	For exclusion diagnosis	Anderson 2017 [27]
Lip biopsy		SSFN	For specific diagnosis of SFN	Morozumi 2008 [50]
Nerve conduction study	Terminal Latency (msec/cm),	SSFN	For exclusion diagnosis (Themistocleous 2014) [16]. These studies often are normal in pure small fiber neuropathies (Hovaguimian 2011) [2].	Saito 2015 [56]
	Compound muscle action potential	SSFN		Saito 2015 [56]
	motor nerve conduction velocity	SSFN		Saito 2015 [56]
	Sensory nerve action potential	SSFN		Saito 2015 [56]
	Sensory nerve conduction velocity	SSFN		Saito 2015 [56]
Nerve biopsy		SFN SCN-SNF	Useful screening (Backonja 2013) [3].	Mishra 2012 [49] Patel 2019 [54]
QSART		Diabetic SFN Autoimmune SFN SSFN	Sudomotor dysfunction may be the earliest manifestation of a distal small fiber neuropathy. Abnormal in >70% of SFN (Low 2006) [17].	Kluding 2012 [45] Liu 2018 [46] Parambil 2010 [53]
Scales	McGill scores	SFN related to CRPS BMS related SFN	Several scales are not specific for SFN. Neuropathy Impairment Score and MNDS demonstrated a weak but significant association with the QSART in the foot, which is a measure of SFN (Zilliox 2016) [23].	Aradillas 2015 [29] Walega 2014 [63]
	MNSI	Diabetic SFN		Kluding 2012 [45]
	Modified Rankin Scale	SS		Pereira 2016 [55]
	Neuropathy Impairment Score	Diabetic SFN Amyloid SNF SFN		Apfel 2000 [28] Keohane 2017 [44] Windebank 2004 [65]
	Neuropathic pain scale	Diabetic SFN Diabetic SNF		Hong 2013 [43] Weintraub 2009 [65]
	Neuropathy Symptom and Change questionnaire	Diabetic SFN		Apfel 2000 [28]
	Numerical rating scale	SFN related to CRPS SS		Aradillas 2015 [29] Birnbaum 2018 [31]
	Patient Benefit Questionnaire	Diabetic SFN		Apfel 2000 [28]
	PGIC	Autoimmune SFN BMS related SFN Diabetic SNF		Liu 2018 [46] Walega 2014 [63] Weintraub 2009 [65]
	SFNSL	SSFN		van Velzen 2014
	Visual analogue scale	Diabetic SFN Diabetic SFN SSFN Keloid related SFN SSFN Diabetic SNF SFN		Hong 2013 [43] Kluding 2012 [45] Morozumi 2008 [50] Uyesugi 2010 [60] van Velzen 2014 [61] Weintraub 2009 [65] Windebank 2004 [65]
	VRS	BMS related SFN		Walega 2014 [63]
	11-point numeric scale	Autoimmune SFN		Liu 2018 [46]
Skin biopsy		SFN related to CRPS SS SFN related to aquagenic pruritus Idiopathic SFN Amyloid SNF Diabetic SFN SFN SFN SFN SSFN SSFN Diabetic SFN SSFN SSFN SSFN SFN variant of Guillain-Barre syndrome	The sensitivity (78–92%) and specificity (65–90%) of skin biopsy for diagnosing a SFN is high (Hovaguimian 2011) [2].	Aradillas 2015 [29] Birnbaum 2018 [31] Cao 2015 [32] Dabby 2006 [33] Keohane 2017 [44] Kluding 2012 [45] MacDonald 2019 [47] Maino 2017 [48] Mishra 2012 [49] Parambil 2010 [53] Saito 2015 [56] Smith 2006 [58] Tavee 2016 [59] van Velzen 2014 [61] Wakasugi 2009 [62] Yuki 2018 [66]
Sudoscan		CIDP SNP	Sudomotor dysfunction is often an early symptom of the SFN.	Nevoret 2014 [52]
Symptoms	Pain	SCN9A-associated SFN SS Prediabetic SFN Fabry related SFN Idiopathic SFN and diabetic SFN SSFN Diabetic SFN	The symptoms are the first guide for the diagnosis and for the choice of the diagnostic program.	De Greef 2016 [34,35] Gaillet 2019 [38] González-Duarte 2015 [39] Hilz 2004 [40] Hoeijmakers 2016 [41] Hoitsma 2006 [42] Kluding 2012 [45]
	Altered temperature sensation	SCN9A-associated SFN Anti-GQ1b antibodies associated with SFN		De Greef 2016 [34,35] Favoni 2018 [37]
	Autonomic dysfunction	idiopathic SFN and diabetic SFN SSFN		Hoeijmakers 2016 [41] Hoitsma 2006 [42]
Threshold	VPT	Diabetic SFN Idiopathic SFN	It could be a useful screening test for small and large fiber neuropathies (Backonja 2013) [3]. It is unable to distinguish between central and peripherical disorders and feigned and true loss of sensation and, moreover it requires the collaboration and conscious integration from the patient (Freeman 2003) [4].	Azmi 2015 [30] Dabby 2006 [33]
	CT	Diabetic SFN Idiopathic SFN SFN Fabry disease related SFN SSFN		Azmi 2015 [30] Dabby 2006 [33] Namer 2019 [51] Schiffmann 2006 [57] van Velzen 2014 [61]
	HP	Diabetic SFN		Azmi 2015 [30]
	WT	Diabetic SFN Idiopathic SFN SFN Fabry disease related SFN SSFN		Azmi 2015 [30] Dabby 2006 [33] Namer 2019 [51] Schiffmann 2006 [57] van Velzen 2014 [61]
	DB-HRV	Diabetic SFN		Azmi 2015 [30]
	IENFD	Diabetic SFN		Azmi 2015 [30]
	CNFD	Diabetic SFN		Azmi 2015 [30]
	CNFL	Diabetic SFN		Azmi 2015 [30]

Small-fiber neuropathies (SFN), Sjögren’s syndrome (SS), sarcoidosis-associated small fiber neuropathy (SSFN), chronic inflammatory demyelinating polyneuropathy (CIDP), burning mouth syndrome (BMS), vibration perception threshold (VPT), cold threshold (CT), heat–pain perception thresholds (HP), warm threshold (WT), deep breathing heart rate variability (DB-HRV), intraepidermal nerve fiber density (IENFD), corneal nerve fiber density (CNFD), corneal nerve branch density (CNBD), corneal Nerve fiber Length (CNFL), sudomotor axon reflex testing (QSART), Michigan diabetic neuropathy screening instrument (MNSI), patient’s global impression of change (PGIC), small-fiber neuropathy screening list (SFNSL), quantitative sensory testing (QST), verbal rating scale (VRS).
